# The impact of four dominating variants and vaccine coverage on Covid-19 mortality: the Malta case: Sarah Cuschieri

**DOI:** 10.1093/eurpub/ckac131.042

**Published:** 2022-10-25

**Authors:** S Cuschieri

**Affiliations:** Anatomy, University of Malta, Msida, Malta

## Abstract

**Background:**

Disease burden can be quantified by mortality. The European Islands of Malta experienced a seesaw of Covid-19 surges in mortality and cases across the pandemic. The study aims to assess the impact of Covid-19 mortality across the four phases dominated by different variants while considering the vaccination coverage among the Malta population.

**Methods:**

Covid-19 epidemiological data was obtained up till end of February 2022 from the websites of the Malta Ministry of Health and the European Centre for Disease Prevention and Control. Data was categorised into the four periods according to reported dominant Covid-19 variant. The Years of life lost (YLL) and Case-Fatality-Ratio (CFR) for each period were estimated. Correlations were performed between mortality and vaccinated age-groups.

**Results:**

The original Covid-19 period (54 weeks) had the highest YLL (6633.53), followed by the Omicron variant period (12 weeks; 2,692.17). The Alpha variant period (7 weeks) had the highest CFR (1.89) followed by the Original Covid-19 (1.37). A significant negative correlation was present between two dose vaccination and the 60-69 years (p = 0.01), 70-79 years (p = <0.01), and 80+ years (p = <0.01) age groups, while a positive correlation was present between the booster dose and the 60-69 years (p = 0.01) age group.

**Conclusions:**

Covid-19 variant’s infectivity, transmissibility, and the effectiveness of the vaccine against the variant play an important role in the ultimate outcome. Reducing mortality by embracing mass vaccination that targets current variants along with other non-pharmaceutical interventions remains paramount.

**Key messages:**

• Mortality is an indicator for assessing the burden of an emerging variant within a population.

• The effectiveness of vaccination against emerging variants plays a role in reducing mortality rates.

